# Diagnosing Ulcerative Colitis: Should We Go Beyond the Surface?

**DOI:** 10.3390/jcm14113690

**Published:** 2025-05-24

**Authors:** Vincenzo Villanacci, Giovanni Maconi, Lucrezia Laschi, Gabrio Bassotti

**Affiliations:** 1 Institute of Pathology, ASST Spedali Civili and University of Brescia, 25123 Brescia, Italy; villanac@alice.it; 2 Gastroenterology Unit, Department of Biomedical and Clinical Sciences, “L.Sacco” Hospital, 20157 Milan, Italy; giovanni.maconi@unimi.it; 3 Pathology Section, Oncology Department, San Giovanni di Dio Hospital, 50143 Florence, Italy; lucrezia.laschi@uslcentro.toscana.it; 4 Gastroenterology and Hepatology Section, Department of Medicine and Surgery, University of Perugia, 06132 Perugia, Italy

**Keywords:** histology, fibrosis, mucosal healing, transmural extension, ulcerative colitis

## Abstract

Ulcerative colitis is a chronic inflammatory bowel disease characterized by continuous mucosal inflammation of the large bowel. However, by conducting a literature search, it emerges that, although being considered a primary mucosal disorder in a subset of patients, the inflammatory process may extend beyond the mucosal surface. For this reason, we reviewed the pertinent literature to evaluate the evidence related to the aforementioned topic. The literature analysis confirmed that, although ulcerative colitis has to be defined as a primary mucosal disease due to its consistent mucosal onset, it can involve deeper layers of the colonic wall. The inefficacy of anti-inflammatory therapies in a considerable proportion of patients, along with the lack of histologic healing and the persistence of inflammatory status and colonic wall thickening at imaging despite mucosal healing, has led to consider an extension of the disease process beyond the mucosal layer. The recent application of more accurate diagnostic tools, both histological and radiological (i.e., intestinal ultrasound and magnetic resonance), has the potential to underline the early signs of disease extension and progression in order to improve ulcerative colitis clinical management.

## 1. Introduction

**Those who go beneath the surface do so at their peril** (Oscar Wilde, 1891)

Ulcerative colitis (UC) is a chronic inflammatory bowel disease (IBD) characterized by a continuous, superficial inflammation confined to the colonic mucosa, with a relapsing–remitting clinical course [1]. This contrasts sharply with Crohn’s disease (CD), which is typified by a discontinuous, transmural inflammation that may affect any segment of the gastrointestinal (GI) tract, from the mouth to the anus, often extending beyond the mucosal layer to involve deeper intestinal wall structures [2].

Historically, UC has been conceptualized as a purely mucosal disorder, a paradigm supported in the time course by its endoscopic and histopathological hallmarks [1,3]. Thus, the diagnosis and follow-up of patients with UC have traditionally relied on the endoscopic assessment and histological evaluation of biopsy samples obtained from the large bowel mucosa. However, in the last years, some emerging evidence has prompted a reevaluation of this longstanding dogma, suggesting that UC may have, in certain clinical contexts, an increased risk of progression, defined as changes of disease phenotype, through transmural effects on the bowel wall [2,3]. This shift in perspective mainly relies on the infrequent pathological findings observed in some patients with longstanding disease [4] and, in particular, on newer non-invasive diagnostic modalities. The latter, especially intestinal ultrasound (IUS), have repeatedly demonstrated that patients with UC featuring moderate-to-severe inflammatory activity frequently exhibit bowel wall thickening that correlates with endoscopic findings and may reflect deeper mural changes [5,6].

These growing observations have thus led researchers to hypothesize that the progression of UC might involve mechanisms traditionally associated with transmural pathology, potentially influencing disease behavior, complications, and therapeutic responses [3]. Furthermore, the histological diagnosis of UC has traditionally relied on mucosal biopsies, which, obviously sampling only the large bowel surface, could be somewhat considered a limiting factor. This debate underscores the need to reconcile the emerging imaging modalities with foundational histopathological criteria [3,6]. Thus, based on the above considerations, it would appear that limiting the pathological assessment of patients with UC to the mucosa could be reputed as restrictive. However, we believe that the concept of transmural disease in this context may be somewhat inappropriate and possibly the result of a misinterpretation of available evidence.

Therefore, the purpose of the present article is to discuss and clarify the concept of histological diagnosis of UC from a primary point of view, with a focus on its histopathological perspectives, also considering the putative aspects of the so-called “transmural” involvement by synthesizing evidence from pathology [4], imaging studies [5,6], and clinical insights into disease progression [3].

## 2. Methods

A comprehensive search of the electronic databases Medline and Science Citation Index was made using the keywords “inflammatory bowel diseases”, “ulcerative colitis”, “colonic fibrosis”, “transmural fibrosis”, “transmural involvement”, “histopathology”, “intestinal ultrasound”, “intestinal magnetic resonance”, and “endoscopy”, in various combinations with the Boolean operators and, or, and not. Only articles related to human studies were included, quoting experimental animal ones only when strictly needed, and manual cross-referencing was performed. Articles published in English between January 1970 and April 2025 were selected, but a search in non-English languages and books was also performed in our Universities and other libraries.

**Clarifying the definitions**

As a starting point, we believe that it is of paramount importance to clearly define the concept under discussion. From this perspective, our view is that UC strongly necessitates a diagnostic framework that distinctly differentiates its peculiar pathological features from the secondary abnormalities that emerge over time as a result of disease chronicity and therapeutic interventions. This distinction is especially critical, as long-term mucosal injury, pharmacological effects, and adaptive responses may obscure or modify the histological landscape, thus complicating retrospective diagnostic assessments. However, the diagnostic paradigm for UC is still to be kept strictly anchored to a careful evaluation of the colonic mucosa from both an endoscopic and histological point of view, with an emphasis on its cardinal histological features ranked by their diagnostic significance.

Concerning this latter point, we feel that the more important morphological/histological elements for the diagnosis of UC in order of importance and relevance are as follows: (1) an increased inflammatory infiltrate; (2) the presence of basal plasmacytosis; (3) the presence of eosinophils intermingled with basal plasma cells; and (4) crypt architectural distortion. Notably, while crypt distortion has traditionally been emphasized, it has also been demonstrated that its diagnostic reliability is limited by technical artifacts, such as tangential sectioning during sample preparation, which may mimic pathological changes [7]. Consequently, when taking into account the guidelines of the European Crohn’s and Colitis Organisation (ECCO) on histopathology of IBD [8], we believe that there are two important points on which the pathologist should focus: (1) inflammatory infiltrate, instead of crypt architectural distortion; (2) within the inflammatory infiltrate, apart from neutrophils whose presence/absence is related to the activity or inactivity of the disease, the presence of basal plasmacytosis intermingled with eosinophils, which must be considered a diagnostic supporter, especially when dealing with a very early onset of UC [9,10,11].

Of note, the four pathological components mentioned above are all located in the context of the mucosa, and this, being closely in contact with the lumen of the colon, likely represents the starting site of the disease. This concept is supported by several pathogenetic theories suggesting that the onset of UC follows an inappropriate immune activation based on the interaction between the host and the intestinal microbiota [12,13,14]. Under this light, recent studies particularly highlight the role of disrupted epithelial barrier function, autophagy, and microbiota-derived metabolites in perpetuating inflammation [12]. Subramanian et al. further delineate how autophagic dysfunction in Paneth cells exacerbates mucosal injury by impairing pathogen clearance and amplifying the release of inflammatory cytokines [14]. Thus, unlike CD, which invariably manifests transmurally, UC is still to be considered a primary colonic mucosal disease [15].

The above considerations and the anatomic specificity have important implications and consequent therapeutic strategies. In fact, topical and/or oral mesalamine, a treatment acting only on colonic mucosa, remains the mainstay therapy of mild to moderately active UC (and not CD), although its effects on the healing process and the degree of histological healing of the large bowel mucosa remain poorly investigated [16]. At present, the main goal of therapy in patients with UC is to achieve a so-called “mucosal healing” or, better, a “histological mucosal healing”, an endpoint considered superior to endoscopic remission in predicting the long-term therapeutic outcomes [17,18]. Histological mucosal healing, in particular (notwithstanding the difficulties in standardizing the histological approach [19,20]), is nowadays considered a crucial target in the treatment of UC [21]. This is due to the fact that there is substantial evidence in the literature demonstrating that patients in whom a histological remission has been achieved display a substantially lower risk of clinical relapse compared to those featuring persistent active inflammation [22].

As biologic therapies advance and new, more targeted drugs for IBD are placed on the market and become commercially available, a precise histopathological characterization will remain central to personalized management. From this perspective, the development and assessment of further technical pathological advances may also result in a useful and improved diagnostic approach to the evaluation of UC. For example, there is recent literature evidence that claudin-2 immunohistochemistry, a tight-junction protein that correlates with neutrophilic infiltration and epithelial permeability, facilitates the assessment of histological mucosal healing because of its expression only in active areas, which also allows the gradation in extension and intensity of activity [23]. Moreover, this assessment also allows us to identify in a relatively easy manner even small foci of residual inflammatory activity after therapy [23].

**Beyond the surface…**

Although, as stated above, UC is classically characterized as a chronic IBD primarily affecting the mucosal layer of the colon, there is also emerging evidence that, as always occurs in nature, there are exceptions. These latter can lead, in the time course (but sometimes even precociously, see below), from an initial limited mucosal inflammation to pathological extensions beyond this superficial layer, challenging the traditional mucosal-centric paradigm. For instance, when there is extensive mucosal ulceration, it is inevitable that the active inflammation will extend into the submucosa and even deeper into the bowel wall, as it invariably occurs in the case of acute fulminant UC with a toxic megacolon. This transmural involvement, though atypical, highlights the dynamic nature of the disease in advanced stages [24].

**Figure 1:**
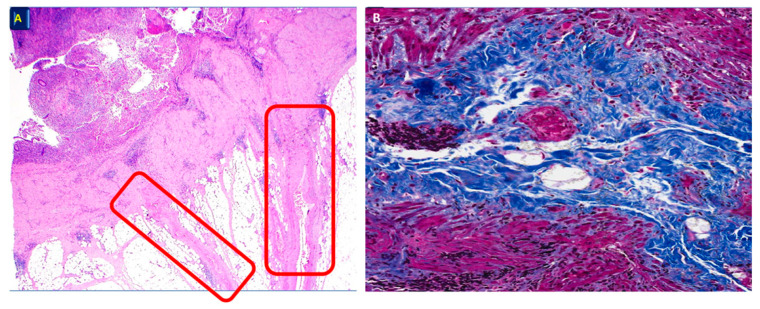
Surgical specimen of UC. (**A**) Extension of the inflammation and consequent. Fibrosis (red rectangle) in the different layers of the bowel wall. H&E, ×3. (**B**) Higher magnification of an area in the right red rectangle, showing extensive fibrosis of the muscularis propria (Masson trichrome, ×40).

Unlike what is observed in patients with CD, only a minority (about 5%) of patients with UC develop clinically evident manifestations due to histologically documented fibrosis [25]. However, it is worth noting that, as shown by studies investigating colectomy specimens (Figure 1), a greater or lesser degree of fibrosis (often associated with muscularis mucosae thickening and collagen deposition) should be considered a common complication of chronic progressive UC, establishing a direct correlation between the severity and the chronicity of mucosal inflammation and the extent of fibrotic wall remodeling [4,26,27]. It should indeed be noted that the longstanding use of therapies over the years, especially when steroids are employed during the alternation of active and quiescent phases of the disease, can induce intramural fibrosis with subsequent stenosis. This simple consideration often poses problems of differential diagnosis from a clinical, imaging, and histological point of view. It is also important to consider that while in CD, the presence of fibrosis is a stigma of the disease, in the case of UC, this aspect is basically a consequence of the therapy. This concept must always be considered when dealing with a patient with suspected IBD. There are, in fact, many instances in which the clinical picture, but also the surgical specimens, might raise the issue of differential diagnosis between UC and CD, and this especially occurs with inexperienced pathologists. In this regard, in cases of toxic megacolon or scheduled interventions, the presence of penetrating inflammation in the context of the submucosa and the muscular coat often leads to the diagnosis of possible CD only on these bases, with obvious consequences from a surgical point of view, delaying the preparation of a pouch. These simple yet objective considerations must always be kept in mind when treating a patient. Of note, although likely responsible for some clinical manifestations, such as abnormal colonic motility [28] (also possibly due to the extension of inflammation to the enteric nervous system with the involvement of the neural regulatory mechanisms [29]) and stenosis [30], the mechanisms, long-term consequences, and therapeutic targeting of fibrosis in UC still remain inadequately unexplored [31].

**…and resurfacing**

Compared to controls, mucosal samples of patients with UC show a persisting dysregulation of fibrosis-associated mediators, as repeatedly observed in both endoscopically healed and active patients [32,33,34]. This fact could, at least in part, justify the persistence of symptoms in a subset of patients, notwithstanding the demonstration of an apparent, visually complete recovery at endoscopy [35]. However, it is still unclear whether these persistent abnormalities may be linked to the lack of histological healing, which is much more difficult to obtain [36]. In fact, it is now evident and well documented in the literature that incomplete histological healing after therapy is associated with a worse clinical outcome and a higher likelihood of relapses [22,37]. Thus, once again, it seems plausible that it is the mucosal damage, originating in the epithelium and lamina propria where there is the onset of the disease, that serves as the primary inducer of the downstream pathological sequelae, including fibrosis appearance.

Under this light, it is of interest that the fibrotic effects on the large bowel wall may also occur with acute inflammation and persist over time despite mucosal healing [3,24]. This observation would justify the rationale for an early aggressive diagnostic and therapeutic approach [38,39,40] in order to abate the initial inflammatory mucosal status and prevent the release of mediators responsible for further damage to the colonic wall. Unfortunately, the current clinical tools available for assessing intestinal fibrosis in patients with UC remain quite limited. For instance, as also emphasized by Krugliak Cleveland et al., the available conventional endoscopic measures fail to capture submucosal or muscularis propria fibrosis, highlighting the need for non-invasive, layer-specific imaging modalities [41].

Detecting fibrosis with diagnostic imaging in UC is challenging due to its superficial nature and the lack of dramatic wall thickening or mass formation, unlike CD, which invariably features deep transmural inflammation that subsequently and frequently causes severe fibrosis, strictures, and even (sub-)occlusion, inflammatory activity in UC is typically limited to the mucosa and submucosa of the large bowel. Consequently, the eventual presence of fibrosis in this condition usually tends to be mild to moderate, diffuse rather than localized, and rarely associated with strictures.

A persistent inflammatory state in UC can also result in an excess collagen deposition, which alters the compliance and the elastic properties of the colonic wall. This, in turn, may affect and impair colorectal motility in different modalities [42,43,44] and contribute to the appearance of symptoms such as diarrhea, urgency, incomplete evacuation, incontinence, and tenesmus.

Thus, in order to face and overcome these challenges, new non-invasive imaging modalities have been introduced in the time course to detect fibrosis or identify fibrosis-related functional changes. The usefulness of these imaging modalities has been primarily investigated in CD to assess the nature of strictures and explore the causes of therapeutic responses, while their application in UC has been more limited, mostly confined to patients with longstanding disease, to evaluate causes of refractoriness and disease activity. However, some data on patients with UC have been obtained, and further studies are ongoing.

For instance, radiomic features derived from computed tomographic images (bowel wall and mesenteric adipose tissue) were recently shown as useful to predict colonic fibrosis and treatment response to biologic drugs in patients with chronic UC [45].

Magnetic Resonance Enterography (MRE) has also proved useful in IBD for detecting chronic wall changes, since it has been nowadays recognized as a powerful, non-invasive tool for assessing bowel fibrosis. Recent advances, such as magnetization transfer imaging (sensitive to the presence of collagen) and native T1 mapping (a quantitative tool for the identification of fibrotic features), enhance its diagnostic capabilities. Diffusion-weighted imaging (DWI), which measures water molecule diffusion using the apparent diffusion coefficient (ADC), showed a strong correlation with histological fibrosis. In fact, ADC has demonstrated 72% sensitivity and 94% specificity in distinguishing fibrotic tissue. Further refinement has been achieved by means of diffusion kurtosis imaging, which assesses tissue diffusional heterogeneity and has shown 95.9% sensitivity in distinguishing mild to absent fibrosis in moderate–severe forms of intestinal inflammation. Another emerging MRE-based approach is based on the use of very small superparamagnetic iron oxide nanoparticles (VSOPs) as contrast agents to detect intestinal inflammation and extracellular matrix changes [46,47,48,49]. Although currently more commonly employed in CD, these innovations hold significant promise for improving fibrosis assessment in UC as well.

**Figure 2:**
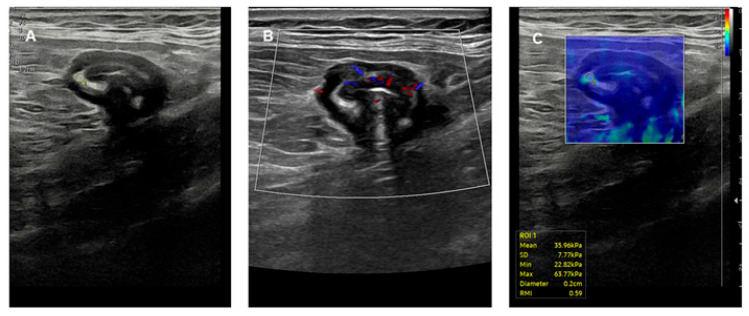
Longstanding left-sided ulcerative colitis under treatment with vedolizumab and in clinical remission in a 70-year-old female patient. Intestinal ultrasound showed a thickened bowel wall of the sigmoid colon with partially preserved stratification at B-mode evaluation (**A**), mild vascularization at color Doppler (**B**), and stiff wall at share wave elastography (**C**), suggesting potential coexistence of fibrosis.

Intestinal ultrasound (IUS), thanks to its high resolution and multiparametric assessment, is also effective in evaluating wall thickening, distinguishing the different wall layers, and assessing the stiffness and vascularization of the colonic wall (Figure 2). When combined with elastography techniques such as strain and shear wave elastography (SWE), IUS provides valuable insights into tissue stiffness fibrotic remodeling. Being radiation-free, non-invasive, repeatable, and relatively inexpensive, this technique appears particularly suitable for the chronic monitoring of patients with UC. In fact, sonoelastography is gaining recognition for its ability to differentiate inflammation from fibrosis (a crucial issue for treatment decisions), to monitor the stiffness of the bowel wall over time, and to help determine whether symptoms may stem from a reversible inflammation or irreversible fibrosis. Strain elastography provides color-coded qualitative stiffness maps and is more operator-dependent, while SWE provides quantitative measurements (in kPa), which are more reproducible and objective. Early studies have reported a sensitivity of 70–85% and specificity of 80–90% compared to histological findings.

In particular, a study by Zhu et al. evaluated 56 consecutive patients with UC who had proctocolectomy and analyzed 112 surgical specimens. The study compared bowel ultrasound activity using the Milan ultrasound criteria [MUC] and the stiffness of the bowel wall by means of strain elastography, whereas colonic fibrosis and inflammation were histologically assessed employing the Geboes score. The study showed that the thickness of the muscularis mucosa, but not that of the bowel wall, was significantly higher in patients with moderate–severe fibrosis than in those featuring none–mild fibrosis. Both colonic wall stiffness and IUS activity assessed by strain ratios and MUC were significantly higher in involved than non-involved segments, but only strain ratios significantly correlated with fibrosis score. The mean strain ratio between the bowel wall and the surrounding bowel tissue had an area under the curve (AUC) of 0.828 for predicting moderate–severe fibrosis [50]. Notably, three resected non-inflamed segments showed the presence of mild fibrosis, indicating the irreversible nature of the fibrotic components despite the recovery from the inflammation. Another study by Yamada et al. investigated the correlation of SWE and shear wave dispersion (SWD) with the activity of UC. The findings of this study showed that SWE values were negatively correlated with UC activity scores (Lichtiger index: rs = −0.404 and UCEIS: rs = −0.506) and that SWD values had no significant correlation (rs ≈ 0), suggesting that SWE may be more relevant than SWD for fibrosis detection in UC [51].

A preliminary investigation, carried out by means of transrectal strain elastography in 30 patients with CD, 25 with UC, and 28 controls with non-inflammatory bowel disease reported that patients with active CD had significantly higher strain ratios compared to patients with active UC, underscoring differences in fibrosis profiles across IBD types [52]. Should these findings be confirmed in more consistent cohorts of patients, the results might have relevant implications concerning the therapeutic response and the outcome in patients with UC, especially concerning the prevention of long-term complications.

## 3. Future Perspectives

The actual lack of therapies able to resolve completely the inflammatory state of the large bowel mucosa in many patients with UC often causes an unavoidable pathological evolution toward fibrosis in the time course. This inadequacy frequently results in a pathological progression over time, wherein further abnormalities extend the borders of the initial ones beyond the mucosa and submucosa, leading sooner or later to thickening and fibrosis of the colonic wall. Such changes are likely implicated in the persistence of some symptoms frequently experienced by patients even after receiving adequate treatments.

In addition, another possible confounding factor is the potential misclassification of some of these patients as having “indeterminate colitis” [53], a term that should only be used when dealing with surgical specimens [54]. It is common experience that this mislabeling often occurs due to a lack of definitive diagnostic criteria, and it is usually re-evaluated and reclassified by expert pathologists upon further examination [55]. Of course, it would be of paramount importance if the pathologist, when assessing a patient for suspected UC, had access to some mucosal markers that could predict or at least suggest the presence of fibrosis deep within the colonic wall.

In response to the above considerations, several efforts are being made to identify the possible broader colonic involvement in patients with UC, especially in those who exhibit resistance to the therapeutic approaches or continue to present with persistent symptoms despite therapy. Of course, this identification should be possibly carried out by means of non-invasive methods, such as IUS [5] and magnetic resonance [56], where available. In this context, IUS has demonstrated significant clinical utility [57,58] and, due to its greater accessibility and versatility (and, last but not least, includes as a bonus the possibility to be used at bedside) compared to magnetic resonance imaging, it could soon emerge as a potentially ideal tool for pursuing this diagnostic [59] (but also prognostic [60]) approach and be conveniently used also for the follow-up [61] of patients with UC. Concerning this latter point, it is worth remembering that IUS in UC also has important further indications, especially concerning high-risk or fragile patients, such as the children population [62] and pregnant women [63].

What does the future hold for us concerning this topic? Although at present, unfortunately, we still have relatively little knowledge of the mechanisms responsible for the “extravasation” of inflammation from the mucosal layer of patients with UC, it is comforting that some interests in this area are being developed [4,38]. Studies on the mechanisms of fibrosis in patients with UC have highlighted the importance of pro-fibrogenic and anti-fibrogenic endogenous factors. Among the former, one of the most important has been identified in the transforming growth factor beta [64], and strategies interfering with its expression and activation are being developed [65]. Similar considerations can be made for interleukin-13 [66,67]. Although to date no effective therapy for fibrosis in UC is available [67,68], new perspectives by exploring other possibilities are being exploited [69], and some experimental animal studies show promising results in the use of anti-fibrotic drugs in this setting [70].

Of interest, the research area is also expanding to the pediatric population. Recent preliminary evidence has suggested that children with refractory disease feature colorectal submucosal fibrosis, correlated with the presence, chronicity, and degree of mucosal inflammation, with the overall fibrosis burden associated with prior anti-tumor necrosis factor use [71]. Moreover, the use of animal models may contribute to a better understanding of the inflammatory mechanisms on the pathophysiology of colonic dynamics during the induction of fibrosis [72,73]. Finally, research priorities are being proposed to individuate the factors underlying and responsible for the damage-related fibrotic progression of this condition [38]. Hopefully, these priorities will lead to a better understanding and, possibly, treatment of these subsets of patients with UC.

## 4. Conclusions

Once again, it must be stressed that UC is primarily a mucosal disease, with an onset limited to the superficial colonic layers, and this is the basic diagnostic aspect that should be identified as soon as possible. Obtaining an early diagnosis implies that treatments may be carried out rapidly, with the ultimate goal of achieving a deep remission that includes complete histological healing of the colonic mucosa (hopefully with the help of new histopathological markers [74,75]) before the pathological processes involve deeper layers of the large bowel.

## Data Availability

No new data were generated or analyzed for this Ms.

## Abbreviations

The following abbreviations are used in this manuscript:

ADC	apparent diffusion coefficient
AUC	area under the curve
CD	Crohn’s disease
DWI	diffusion-weighted imaging
ECCO	European Crohn’s and Colitis Organisation
GI	gastrointestinal
IBD	inflammatory bowel diseases
IUS	intestinal ultrasound
MRE	magnetic resonance enterography
MUS	Milan ultrasound criteria
SWD	shear wave dispersion
SWE	shear wave elastography
UC	ulcerative colitis
VSOPs	very small superparamagnetic iron oxide nanoparticles
